# Systems Modeling Identifies Divergent Receptor Tyrosine Kinase Reprogramming to MAPK Pathway Inhibition

**DOI:** 10.1007/s12195-018-0542-y

**Published:** 2018-07-26

**Authors:** Allison M. Claas, Lyla Atta, Simon Gordonov, Aaron S. Meyer, Douglas A. Lauffenburger

**Affiliations:** 1 Department of Biological Engineering, Massachusetts Institute of Technology, 77 Massachusetts Avenue, Cambridge, MA 02139 USA; 2 Department of Bioengineering, Jonsson Comprehensive Cancer Center, Eli and Edythe Broad Center of Regenerative Medicine and Stem Cell Research, University of California, Los Angeles, Los Angeles, CA 90095 USA

**Keywords:** Triple negative breast cancer, EGFR, Her2, Met, Axl, Mek inhibition, Erk inhibition, BET inhibition

## Abstract

**Introduction:**

Targeted cancer therapeutics have demonstrated more limited clinical efficacy than anticipated, due to both intrinsic and acquired drug resistance. Underlying mechanisms have been largely attributed to genetic changes, but a substantial proportion of resistance observations remain unexplained by genomic properties. Emerging evidence shows that receptor tyrosine kinase (RTK) reprogramming is a major alternative process causing targeted drug resistance, separate from genetic alterations. Hence, the contributions of mechanisms leading to this process need to be more rigorously assessed.

**Methods:**

To parse contributions of multiple mechanisms to RTK reprogramming, we have developed a quantitative multi-receptor and multi-mechanistic experimental framework and kinetic model.

**Results:**

We find that RTK reprogramming mechanisms are disparate among RTKs and nodes of intervention in the MAPK pathway. Mek inhibition induces increased Axl and Her2 levels in triple negative breast cancer (TNBC) cells while Met and EGFR levels remain unchanged, with Axl and Her2 sharing re-wiring through increased synthesis and differing secondary contributing mechanisms. While three Mek inhibitors exhibited mechanistic similarity, three Erk inhibitors elicited effects different from the Mek inhibitors and from each other, with MAPK pathway target-specific effects correlating with Erk subcellular localization. Furthermore, we find that Mek inhibitor-induced RTK reprogramming occurs through both BET bromodomain dependent and independent mechanisms, motivating combination treatment with BET and Axl inhibition to overcome RTK reprogramming.

**Conclusions:**

Our findings suggest that RTK reprogramming occurs through multiple mechanisms in a MAPK pathway target-specific manner, highlighting the need for comprehensive resistance mechanism profiling strategies during pharmacological development.

**Electronic supplementary material:**

The online version of this article (10.1007/s12195-018-0542-y) contains supplementary material, which is available to authorized users.

## Introduction

Detailed genetic understanding of molecular cancer drivers has enabled the development of targeted cancer therapeutics. Well-characterized cancer targets such as mutant EGFR in non-small cell lung cancer (NSCLC) and the BCR-Abl fusion gene in chronic myelogenous leukemia (CML) led to initial breakthroughs,^12^,^31^,^37^ and success *via* this approach has continued to expand as more than 150 targeted therapeutics have been approved to date by the FDA to treat various cancer subtypes.^48^ Unfortunately, sustained therapeutic efficacy has been limited by the emergence of drug resistance. Enabled by broadening availability of advanced genome sequencing technologies, genetic mechanisms of drug resistance have been widely identified—commonly mutation or amplification in the target itself or alternate proteins.^14^,^35^,^36^,^41^ However, emerging evidence is showing that non-genetic mechanisms also contribute significantly to drug resistance, such that a substantial proportion of resistance cannot be readily attributed to genetic lesions. For instance, target and alternative receptor tyrosine kinases (RTKs) can exhibit enhanced activities *via* increased expression even in the absence of gene amplification,^4^,^10^,^35^,^46^,^49^ including by means of modulated ligand binding and/or receptor oligomerization.^25^,^50^,^52^ Due to the many RTKs that may contribute to resistance, monitoring coordinated changes in RTK networks, termed “RTK reprogramming”, has become important for evaluating cancer drug resistance.^10^,^13^,^45^

While identification of mutation or amplification of the target protein can lead to improved second and third line inhibitors that have advantageous properties, such as alternate binding motifs, covalent binding, or the combination of antibodies and small molecule inhibitors,^26^,^40^ elucidation of additional activated proteins, whether alternative RTKs or downstream signaling molecules, can guide combination treatment with inhibitors against a second target. When gene expression networks are broadly altered, it may be useful to employ epigenetic inhibitors, such as bromodomain and extra-terminal domain (BET) inhibitors, to limit the dynamic response of numerous potential targets simultaneously.^9^

A highly relevant clinical application representing a major unmet treatment need is triple negative breast cancer (TNBC), which is an aggressive disease accounting for approximately 15% of invasive breast cancers and is defined as progesterone receptor (PR) negative, estrogen receptor (ER) negative, and Her2 negative.^38^ Although lacking traditional markers identified in breast cancer, the EGFR inhibitor erlotinib has been shown to have subtype specificity for basal/TNBC.^21^ Furthermore, 37% of patient samples classified as TNBC overexpress EGFR.^38^ However, in a phase II study of Cetuximab for EGFR inhibition in combination with carboplatin for treatment of TNBC, fewer than 20% of patients responded to treatment even though they had EGFR activation prior to treatment. Analysis of pre- and post-treatment biopsy samples found that the EGFR pathway was upregulated in 81% of pre-treatment samples and eight of thirteen patients retained high EGFR pathway expression in the presence of EGFR inhibition, indicating pathway maintenance downstream of EGFR.^5^ As the MAPK pathway is one of the major signal transduction pathways downstream of EGFR that promotes growth and survival, it has been studied for its role in TNBC. In fact, approximately 80% of TNBCs have amplification in EGFR, KRAS, or BRAF proteins, providing a rationale for targeting the MAPK pathway.^27^ Further, TNBC cell lines are preferentially sensitive to Mek inhibition supporting MAPK inhibition in TNBC.^23^

Despite this compelling rationale, pre-clinical and clinical evidence indicates that TNBC cells undergo RTK reprogramming, limiting response to Mek inhibition.^13^,^34^ While RTK reprogramming has been described as a transcriptionally regulated event,^13^,^45^ work from Miller *et al*. demonstrated abrogation of RTK ectodomain shedding as an alternative mechanism.^34^ With multiple competing, or more likely complementary, hypotheses found in different studies, a need is clear for a more integrative perspective on how multiple mechanisms may concomitantly contribute to drug resistance even through a particular phenomenon such as dynamic alterations in RTK levels.

Our work here accordingly aims to develop an integrative framework based on quantitative experimentation and a computational model that quantifies contributions of non-genetic mechanisms to many altered RTK levels in parallel. By leveraging non-specific cell labeling and antibody specific measurements we have developed a methodology that is amenable to systems level characterization and provides robust estimates for parameters that are historically cumbersome to measure directly. We apply this framework in the context of drug resistance to MAPK inhibition in TNBC to clarify the absolute contributions of competing processes. In doing so, we show that Axl and Her2 levels increase following Mek inhibition not only through increased synthesis but also through secondary mechanisms, including decreased protein degradation and endocytosis. Additionally, receptor degradation and endocytosis decreased broadly with only context-specific quantitative effects on RTK levels and decreased proteolytic shedding does not quantitatively contribute to altered cellular RTK levels. Furthermore, we identify differences in the RTK reprogramming response to Mek inhibitors vs. Erk inhibitors. Taken together, we have identified integrative, RTK specific, and MAPK inhibitor-specific RTK reprogramming responses in TNBC.

## Results

### Integrative Model Quantifies Mechanistic Cellular Processes and Basal Cell State

**Figure 1:**
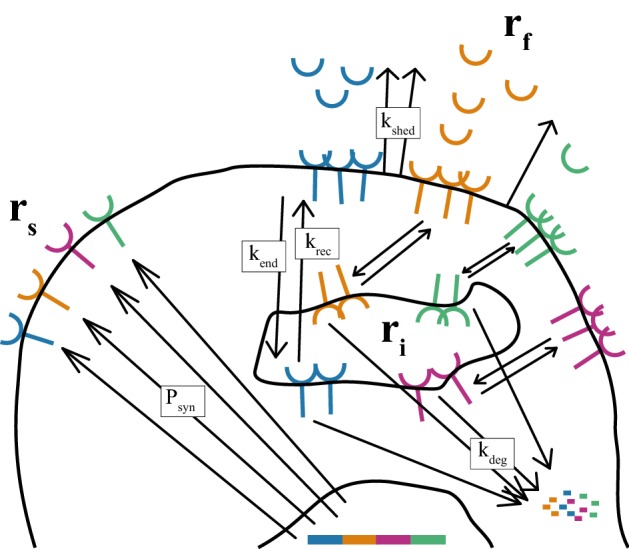
Model schematic. Axl (blue), Met (orange), EGFR (green), and Her2 (purple) receptors exist in one of three compartments: Cell surface (*r*_s_), intracellular (combined endosomal, lysosomal, nuclear) (*r*_i_), or freely circulating in the extracellular medium (*r*_f_). *P*_syn_ represents net protein synthesis, the sum of transcriptional and translational regulation, *k*_deg_ represents protein degradation from the internal compartment, *k*_shed_ represents proteolytic shedding of cell surface extracellular domains, and *k*_end_ and *k*_rec_ represent endocytosis and recycling to and from the internal protein pool (*i.e.,* endosomes) respectively.

We used the model structure depicted in Fig. 1 to describe the mechanistic processes of interest governing protein levels and subcellular localization. Briefly, RTKs are either on the cell surface, intracellular (endosomal/lysosomal/nuclear), or free ectodomains are circulating in the extracellular environment (supernatant). Zeroth order protein synthesis adds protein to the cell surface, while first order rate constants govern transport between the compartments by proteolytic shedding, endocytosis, recycling, and degradation.

**Figure 2:**
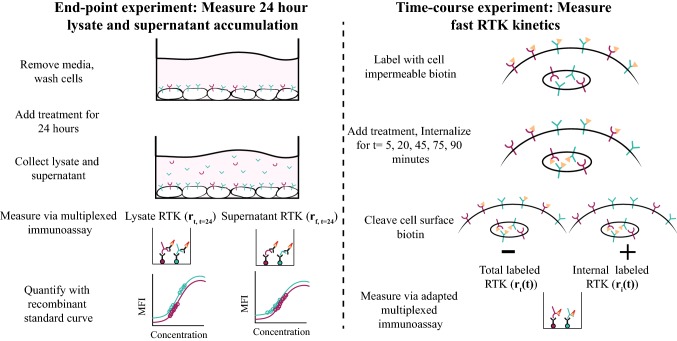
Experimental methodology for measuring varying RTK levels and kinetics. (left) End point experiment workflow whereby after 24-h treatment lysate and supernatant samples are collected and measured by bead-based ELISA with a recombinant protein standard. (right) Time-course experiment workflow whereby cell surface proteins are non-specifically labeled with biotin, labeled proteins are trafficked over varying time points, and surface biotin is either cleaved or un-cleaved. Samples are measured with an adapted bead-based ELISA. Colors represent two proteins measured simultaneously and independently.

To inform the model, both end point and time-course experiments were performed to capture processes manifesting over both fast and slow time scales and provide additional unique model information. As summarized in Fig. 2, end point measurements are made by treating cells for 24 h. Lysate and supernatant samples are quantified with a multi-plexed bead-based ELISA and recombinant protein standard. Time-course measurements are pulse-chase experiments, whereby cell surface proteins are non-specifically labeled with a cleavable, cell impermeable biotin. After varying incubation times from 5 to 90 min to facilitate labeled protein trafficking, cells are either lysed in whole or after the cell surface biotin label is stripped, yielding an internal pool of labeled protein. Samples are then measured by total protein pull-down with primary antibodies in a multi-plexed bead-based ELISA and labeled protein detection with a streptavidin conjugate, permitting relatively straightforward multi-plexing. This technique combines biotinylation non-specificity of binding to essentially all accessible surface proteins with antibody specificity to selectively quantify those proteins of particular interest. Furthermore, the direct labeling and measurement of the protein of interest allows us to characterize the basal cell state in addition to perturbation effects (as opposed to traditional methods dependent on a labelled ligand or antibody binding effects), further increasing the applicability of this methodology.

**Figure 3:**
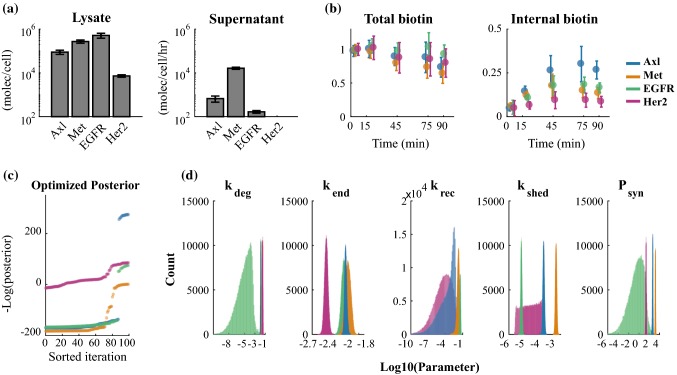
Quantitative experiments and parameter estimation define MDAMB231 cell basal state. (**a**) End point sample quantification for total lysate (left) and supernatant (right) samples for control treated MDAMB231 cells. mean ± standard deviation, *n* = 12. (**b**) Time-course measurement of total biotin labeled RTK (left) and internal biotin labeled RTK (right) samples for control treated MDAMB231 cells. mean ± standard deviation, *n* = 18. (**c**) Optimized posterior value from deterministic *patternsearch* algorithm for 100 start sites determined by latin hypercube sampling and ordered by increasing -log(posterior) values. (**d**) Parameter posterior distributions from adaptive metropolis algorithm. Data are summed from 4 independent chains, 100,000 iterations each, minus 20,000 step burn-in period. *P*_syn_ has units of molecules cell^−1^ min^−1^ and *k*_deg_, *k*_end_, *k*_rec_, and *k*_shed_ have units of min^−1^. Blue—Axl, orange—Met, green—EGFR, purple—Her2.

To characterize the basal cell state of MDAMB231 TNBC cells, we collected end point and time-course measurements for Axl, Met, EGFR, and Her2 in control treated cells (Figs. 3a and 3b). Axl, Met, and EGFR are highly expressed at levels on the order of 10^5^–10^6^ molecules/cell, consistent with values characterized previously for EGFR in cancer cell lines.^44^ We also found high supernatant levels of Axl and Met, ranging from ~ 1 to 6% of lysate levels shed per hour respectively, highlighting the rapid turnover of protein through proteolytic shedding. Furthermore, we observe different RTK kinetics, with biotinylated Met levels turning over the most rapidly and internal Axl levels accumulating to the highest relative level.

We utilized Bayesian statistics to calculate the posterior probability for model parameters, identifying parameter distributions accounting for experimental variability which provides a more comprehensive characterization than single point estimates. Furthermore, we leveraged prior distributions to enable estimation of biologically relevant parameter regimes without over-constraining parameters based on literature values from different proteins, cell lines, or environmental contexts. Parameter estimation was first performed *via* a deterministic direct search algorithm with 100 semi-random start sites identified by latin hypercube sampling (see “Materials and Methods”). While we observe start site dependent local optima for optimized negative log(posterior) values across RTKs, we also observe a convergence to what we presume to be the global minimum posterior value (Fig. 3c). As such, deterministic optimization yields our presumed global optimum parameter set. To address parameter variability and generate distributions as opposed to single point estimates, we used an adaptive metropolis algorithm to generate the full parameter posterior distributions describing the experimental data (Fig. 3d).

Generally, parameter values across RTKs for endocytosis, recycling, degradation and synthesis were consistent with published literature values for EGFR^15^,^17^,^18^,^22^,^39^,^51^ and Axl endocytosis and recycling rates were similar to those estimated from a model of ligand-receptor interaction and trafficking.^33^ In addition to estimated values consistent with literature reports, we find relatively narrow distributions for parameter estimates with the methodology developed here. Compared to traditional trafficking measurement methods that depend on radioactive or fluorescently labeled ligands and treatment with broad-spectrum inhibitors which are often toxic to the cells, we were able to achieve constrained parameter estimates with experiments that are readily accessible, extendable, and avoid using inhibitors that may introduce off-target effects.

Of note, EGFR degradation and synthesis have limited comparability to literature values as a consequence of low identifiability and strong parameter covariation which was not observed with other receptors and treatments. Furthermore, we observe a roughly uniform distribution for Her2 *k*_shed_ although there was no Her2 measured in the supernatant. This is a result of assuming the supernatant measurement can be any value less than the lower limit of quantitation (LLOQ) such that the Her2 *k*_shed_ distributions herein represent biologically plausible shedding rates that would yield un-detectable supernatant levels consistent with our experimental measure. While EGFR kinetics, and Her2 kinetics to a lesser extent, have been well-documented in the literature, we have gained insights here into the basal cellular behavior of the less studied Axl and Met receptors.

**Figure 4:**
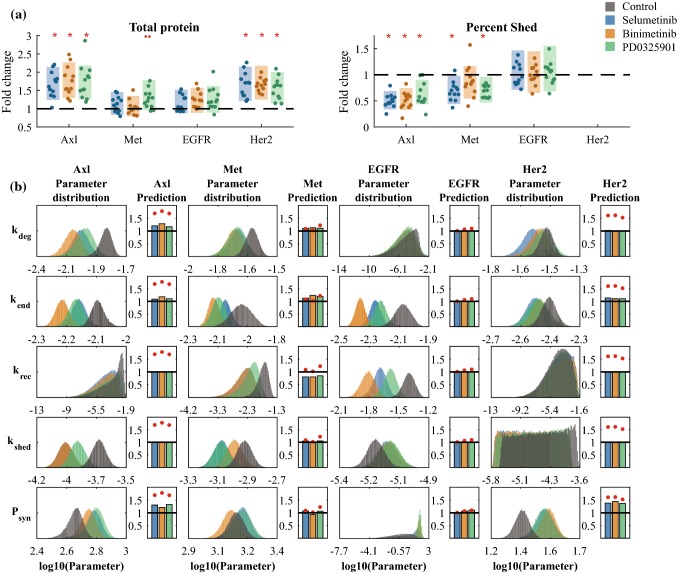
Mek inhibitors increase Axl and Her2 lysate levels and alter multiple parameter estimations. (**a**) (left panel) End point lysate measurements and (right panel) end point percent shed (*r*_f_/*r*_t_) levels normalized to the mean control treated value for MDAMB231 cells treated for 24 h with three Mek inhibitors. Points indicate experimental data (*n* = 12) and shaded bars represent the range of simulated data from resampling 10% of parameter sets. Asterisk indicates *p* < 0.01 and ·· indicates *p* < 0.05 with a two-sample *t* test with Bonferroni multiple hypothesis correction. (**b**) Parameter posterior distributions (summed for 4 independent chains, 100,000 iterations each, 20,000 step burn-in time) for control and Mek inhibitor treated cells and predicted protein level fold change relative to control treatment (red dots indicate observed treatment fold change). *P*_syn_ has units of molecules cell^−1^ min^−1^ and *k*_deg_*, k*_end_*, k*_rec_, and *k*_shed_ have units of min^−1^. Black—control, blue—selumetinib, orange—binimetinib, green—PD0325901.

**Figure 5:**
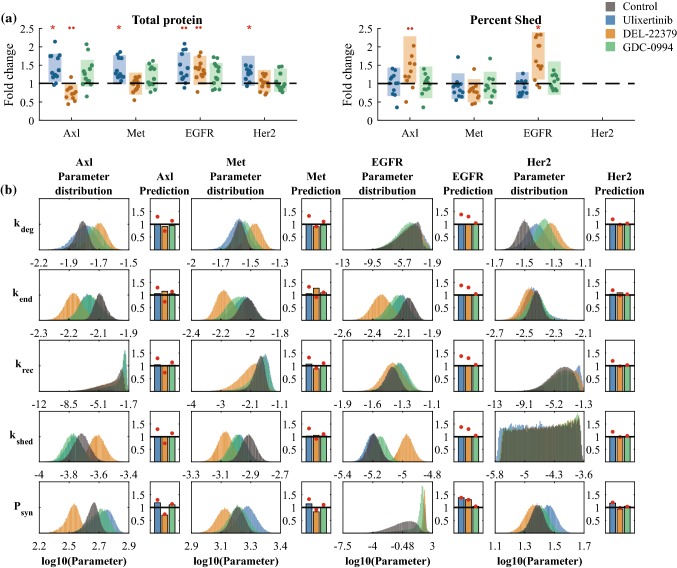
Erk inhibitors have compound dependent parameter changes that vary from Mek inhibition. (**a**) (left panel) End point lysate measurements and (right panel) end point percent shed (*r*_f_/*r*_t_) levels normalized to the mean control treated value for MDAMB231 cells treated for 24 h with three Erk inhibitors. Points indicate experimental data (*n* = 12) and shaded bars represent the range of simulated data from resampling 10% of parameter sets. Asterisk indicates *p* < 0.01 and ·· indicates *p* < 0.05 with a two-sample *t* test with Bonferroni multiple hypothesis correction. (**b**) Parameter posterior distributions (summed for 4 independent chains, 100,000 iterations each, 20,000 step burn-in time) for control and Mek inhibitor treated cells and predicted protein level fold change relative to control treatment (red dots indicate observed treatment fold change). *P*_syn_ has units of molecules cell^−1^ min^−1^ and *k*_deg_*, k*_end_*, k*_rec_, and *k*_shed_ have units of min^−1^. Black—control, blue—ulixertinib, orange—DEL-22379, green—GDC-0994.

To validate the ability of our experimental and computational framework to quantitate cellular mechanistic processes, we used two perturbations with known cellular effects. First, cells were stimulated with EGF which is known to internalize EGFR and Her2 and down-regulate lysate levels.^22^,^39^,^44^ Indeed, EGFR and Her2 lysate levels were decreased after 24 h whereas Axl and Met levels remained unchanged (Supplemental Fig. 1a). Additionally, receptor internalization was increased for EGFR and Her2 and the endocytosis (*k*_end_) parameter distribution was increased for cells stimulated with EGF (Supplemental Figs. 1b, 1c). Second, cells were treated with batimastat, a broad-spectrum metalloprotease inhibitor which has been used to decrease proteolytic shedding of Axl and Met.^34^ Axl and Met lysate levels were increased after 24 h with batimastat treatment with a concomitant decrease in the supernatant levels, consistent with model estimations of decreased *k*_shed_ (Supplemental Fig. 2). For both cases and all other treatments used herein, we find good agreement between experimental data and simulated data generated from randomly sampling 10% of parameter sets (*i.e.,* sampling from adaptive metropolis steps, maintaining parameter covariate relationships) for both end point data (Figs. 4a and 5a, Supplemental Figs. 1a, 2a, 6a, 7a) and time-course data (Supplemental Figs. 1b, 2b, 3, 4). This provides confidence that the parameter posterior distributions on which we draw biological conclusions not only fit the data well but also reflect the underlying sources of variability in the data. Together, these two examples highlight the ability of the methodology to capture and quantify known RTK specific perturbations.

### Mek Inhibitors Alter Axl and Her2 Levels Through Distinct Integrative Mechanisms

Three Mek inhibitors, selumetinib, binimetinib, and PD0325901, were tested for their induction of RTK reprogramming. To allow for compound dependent downstream effects (*i.e.,* viability, RTK reprogramming), we used concentrations that were 100 times greater than the reported *in vitro* IC_50_ values for target inhibition. We find that the three compounds have different IC_50_ values in MDAMB231 cells, although the selected concentration values result in similar inhibitory effects (Supplemental Fig. 5).

After 24-h treatment, MDAMB231 and SUM159 cells had increased Axl and Her2 lysate levels and decreased Axl and Met supernatant accumulation relative to lysate levels (represented as percent shed) (Fig. 4a, Supplemental Fig. 6a). Parameter distributions underlying the treatment induced changes are shown in Fig. 4b and Supplemental Fig. 6b. Looking across RTKs, we observe multiple trends in comparison to control treatment. First, we see a decrease in endocytosis (*k*_end_) across all inhibitors and RTKs. Second, Axl and Met degradation (*k*_deg_) and shedding (*k*_shed_) are decreased across Mek inhibitors and to a greater extent in MDAMB231 cells. Third, Axl and Her2 synthesis (*P*_syn_) are increased across inhibitors. Although the mechanisms described here (endocytosis, degradation, *etc*.) represent fundamental cellular processes, the identification of RTK specific effects indicates underlying molecular details, such as mediator proteins and their abundance, activity, or co-localization, are likely driving the mechanism context dependency.

While changes in broad parameter distributions are informative as to how cellular processes are changing, we wished to quantitate how these changes translated to changes in RTK abundance. To quantitate the effect of treatment-induced parameter distribution changes, lysate levels were predicted using the mean parameter values for control treatment and singly substituting mean parameter values for each treatment. The model therefore allows one to predict the quantitative contribution of individual parameter mean changes relative to the entire treatment induced changes for total protein levels. For Her2, we find that protein synthesis is the dominant driver of increased lysate levels, although decreased endocytosis has secondary contributions as well (Fig. 4b, Supplemental Fig. 6b). Axl, on the other hand, is equally governed by the increased protein synthesis and decreased degradation rates, with smaller contributions from decreased endocytosis. Her2 mRNA levels have been reported to increase with Mek inhibition;^13^ however, Axl levels have not been reported to have significantly altered mRNA levels^13^,^34^ (unpublished in-house data). Two hypotheses are consistent with both pieces of data, the first that mRNA to protein levels do not always correlate well^30^ such that small, statistically insignificant mRNA changes could yield significant protein changes, and the second that protein synthesis control occurs post-transcriptionally.

Although these model predictions are based on mean parameter values, we assessed the contributions of parameter variability to model predictions using 10,000 randomly sampled control and treated parameter sets (Supplemental Fig. 8). We find the distribution of predicted changes for the conclusions drawn above to have little to no overlap with an unchanged value of 1, providing support for the predictive capacity in the presence of parameter variability.

Surprisingly, we saw that the decreases in proteolytic shedding had only quantitatively minor predicted effects on lysate levels in the cell line models tested here, although it has previously been shown to serve as a biomarker for poor patient progression free survival in melanoma patients treated with Mek and Braf inhibitors.^34^ Further study in systems with higher levels of shed protein will be needed to gain a better understanding of if and when proteolytic shedding is a major contributor to drug resistance. Importantly, our result here does not eliminate a potential role of proteolytic shedding in other models and our analysis framework highlights the importance of quantitative modeling for distinguishing between correlative and causative changes. Additionally, our methodology identifies a yet un-studied contributor to Mek inhibitor induced RTK reprogramming, decreased protein degradation and endocytosis.

### Erk Inhibitors Have Compound Dependent Effects that Vary from Mek Inhibitors

To compare the RTK reprogramming effect following inhibition of the MAPK pathway at different points, we similarly tested three Erk inhibitors, ulixertinib, DEL-22379, and GDC-0994. When compared to Mek inhibitors, we see that only one of the Erk inhibitors, ulixertinib, increased Axl and Her2 lysate levels in MDAMB231 cells and had no effect in SUM159 cells (Fig. 5a, Supplemental Fig. 7a). Additionally, ulixertinib increases Met and EGFR levels in MDAMB231 cells, showing differences from Mek inhibitors. These increases are primarily driven by increased synthesis of all RTKs with an absence of decreased protein degradation and endocytosis (Fig. 5b). Interestingly, ulixertinib and GDC-0994 are both Erk TKIs whereas DEL-22379 is an Erk dimer inhibitor. Not only do we see different responses within TKIs, but these responses, as well as Mek inhibitors, are further different from DEL-22379, indicating that variations in Erk dimerization is not driving the observed differences seen here (Fig. 5, Supplemental Fig. 7).

### MAPK Pathway Has Target-Specific RTK Reprogramming

**Figure 6:**
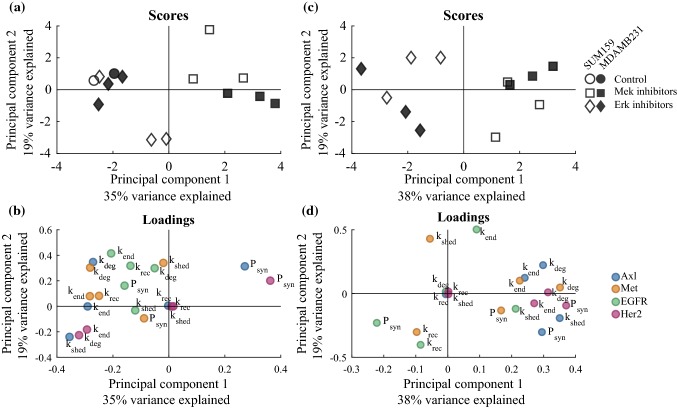
Principal component analysis identifies separation of Mek and Erk inhibitors and cell type specific responses. (**a**) and (**b**) Scores and Loadings plots respectively for PCA analysis where mean parameter values were used as variables. (**c**) and (**d**) Scores and Loadings plots respectively for PCA analysis where predicted protein fold change values were used as variables. In Scores plots, circles indicate control treated samples, squares indicate Mek inhibitor treated samples, and diamonds indicate Erk inhibitor treated samples. Filled symbols indicate MDAMB231 cells while open symbols indicate SUM159 cells. In Loadings plots, variables are color coded by RTK (blue—Axl, orange—Met, green—EGFR, purple—Her2) and mechanistic processes are labeled accordingly.

We utilized principal component analysis (PCA) to identify the greatest variation across cell lines and inhibitors (scores) and the variables (loadings) contributing to these changes. PCA was performed with two different variable definitions: (1) mean parameter values (Figs. 6a and 6b) and (2) predicted protein change (Figs. 6c and 6d). In both cases, there is a clear separation between Mek and Erk treated samples, with Erk inhibitor treated samples more similar to control treated. Axl and Her2 synthesis (*P*_syn_), Axl, Her2 and Met endocytosis (*k*_end_), and Axl, Met, and Her2 degradation (*k*_deg_) are among the loadings of largest magnitude and with directionality consistent with Mek-Erk score separation in both PCA analyses, indicating they are major drivers of the MAPK target specific RTK reprogramming response observed.

We further observe cell type specific effects as a shift from Control/Erk to Mek treatment along principal component 1 for MDAMB231 cells and along principal component 2 for SUM159 cells. Whereas Mek inhibitors alone decrease endocytosis (*k*_end_) in MDAMB231 cells, both Mek and Erk inhibitors decrease endocytosis in SUM159 cells. Alternatively, Met protein synthesis (*P*_syn_) is decreased with Mek inhibition and unaffected with Erk inhibition in SUM159 cells and unaffected with both Mek and Erk inhibition in MDAMB231 cells. Cell specific responses are not surprising, especially considering their varying genetic background (MDAMB231- Kras mutant,^23^ PIK3CA wild type^42^ SUM159- Hras mutant, PIK3CA mutant),^3^ although further study is needed to address contributions of these genetic factors to the varying RTK reprogramming phenotypes observed.

**Figure 7:**
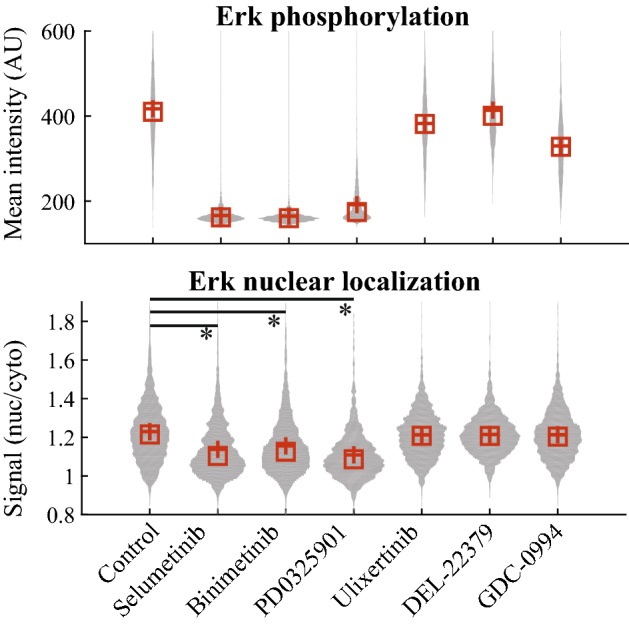
Mek inhibitors decrease Erk phosphorylation and nuclear localization. Quantitation of total phospho-Erk (top) and nuclear/cytoplasmic Erk (bottom) from immunofluorescence imaging of Erk, phospho-Erk (T202/Y204), and DAPI. Crosses indicate distribution mean and squares indicate distribution median. Asterisk indicates *p* < 0.01 by Wilcoxon rank sum test with Bonferroni correction.

Mek and Erk inhibitors had different effects on Erk phosphorylation where Mek inhibitors decreased Erk phosphorylation (T202/Y204) and Erk inhibitors did not (Fig. 7, Supplemental Fig. 9). Two hypotheses for phosphorylation site effects are around Erk dimerization and subcellular localization. As we have already observed protein levels and parameter distributions with an Erk dimer inhibitor, DEL-22379, that vary from those of Mek and Erk TKIs, it is unlikely that differences in Erk dimer formation in a phospho-site dependent manner describe the observed Mek and Erk inhibitor differences. However, Erk nuclear localization is decreased specifically with Mek inhibition (Fig. 7, Supplemental Fig. 9), consistent with the role of Erk phosphorylation on nuclear transport.^7^

### Mek and BET Inhibition Have Differing Effects, Motivating Combination Treatment and Sensitizing Cells to Axl Inhibition

In the case of cellular RTK reprogramming whereby no single alternate target exists for combination treatment, the use of epigenetic regulators to prevent the transcriptional response has been postulated and tested in certain models.^8^,^45^ In TNBC cell lines, Mek induced RTK reprogramming was reported to act through de-stabilization of Myc, inducing transcription of proteins that are normally repressed.^13^ JQ1, a BET bromodomain inhibitor, was developed to inhibit the recognition of acetylated lysine residues by BET family proteins and not only has shown some specificity for Myc driven genes^11^ but other genetic targets as well.^2^,^43^ Additionally, JQ1 was found to be effective at inhibiting viability in TNBC cell lines *in vitro* and *in vivo*^43^ as was previously seen for Mek inhibitors.^23^ Although both Mek and BET inhibition are methods of perturbing Myc genetic regulation it is unclear whether their mechanism of action is redundant and thus is of interest to compare the RTK rewiring response for both treatments alone and in combination. Furthermore, Mek inhibitor increased Her2 levels were primarily driven by protein synthesis while Axl levels were only driven in part. Through combination treatment with Mek and BET inhibition (with the caveat that JQ1 has target specificity that may not include Her2 and Axl), we can test the model predictions for the quantitative synthesis role for Her2 and Axl and expect that combination treatment would return Her2 levels to baseline whereas Axl levels would remain elevated.

**Figure 8:**
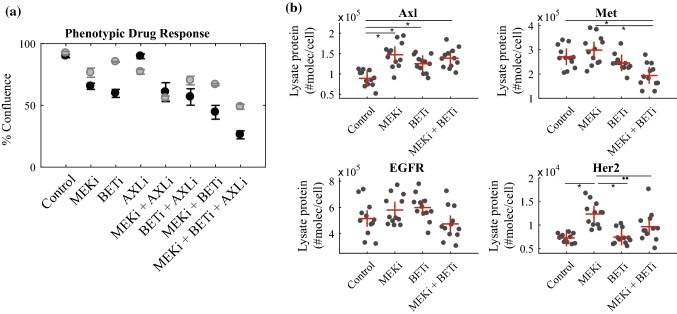
Mek and BET inhibitor combinations improve response and sensitize cells to Axl inhibition. (**a**) Phenotypic drug response for MEKi (selumetinib, 1.5 *μ*M), BETi (JQ1, 0.2 *μ*M), and AXLi (R428, 1 *μ*M) in MDAMB231 cells. Proliferation (black) and Migration (grey) responses, represented by either % confluence of the entire well or of the wound respectively, were measured for varying single and combination treatments. Proliferation measurement was after 100 h and migration measurement was after 24 h. Mean ± standard deviation with *n* = 8. (**b**) Lysate levels in molecules/cell for RTKs when treated with control, MEKi, BETi, or MEKi + BETi in MDAMB231 cells. Points indicate experimental data (*n* = 12) and crosses indicate mean values. Asterisk indicates *p* < 0.01 and ·· indicates *p* < 0.05 determined by pairwise *t* tests with Bonferroni correction.

Whereas both Mek inhibition (selumetinib) and BET inhibition (JQ1) had anti-proliferative and anti-migratory effects alone, combination treatment further inhibited both processes (Fig. 8a). Interestingly, Her2 lysate levels were increased with Mek but not BET inhibition yet the combination of the two reduced Her2 levels back to baseline (Fig. 8b). This finding indicates that the Mek inhibitor-induced Her2 transcription becomes BET dependent whereas basal transcription is not. On the other hand, Mek inhibition and BET inhibition both increased Axl levels, resulting in sustained high Axl protein and phosphorylation levels (data not shown) with combination treatment. Combined, this further sensitized cells to inhibition with R428, an Axl inhibitor, whereby R428 had no anti-proliferative effect in combination with either Mek or BET inhibitor alone but does in the combined background of Mek + BET inhibition. Together, these results not only support the use of Mek and BET inhibitors in combination due to their non-overlapping and context-dependent effects on RTK reprogramming, but further support combination inhibition of Axl in this context.

## Discussion

An integrative, multi-receptor model developed here expands our knowledge of targeted cancer therapy-induced RTK reprogramming by providing mechanistic insights into the underlying processes responsible for changes in RTK levels. Previous work has described the RTK reprogramming phenotype as a transcriptionally regulated event,^13^,^45^ putting the spotlight on BET inhibitors as viable treatment options to prevent resistance onset. Our work additionally identifies the presence of non-synthesis mediated changes in protein levels, the therapeutic implications for which have yet to be explored in greater detail.

We describe divergent RTK reprogramming phenotypes between Mek and Erk inhibitors, indicating that Mek and Erk inhibitors should not be considered interchangeable. Assuming that both Mek and Erk inhibitors decrease Erk kinase activity, differences could be driven by Erk phosphorylation dependent cellular localization, protein binding, or alternate Mek substrates. While we have not assessed differences in Erk binding to target proteins with Mek or Erk inhibitors, we have observed decreased Erk nuclear localization following Mek but not Erk inhibition. In addition to the traditional role of Erk substrate phosphorylation, Erk has been found itself to be associated with chromatin,^24^,^32^ potentially acting as a transcription factor to link subcellular localization and protein synthesis variations with Mek and Erk inhibitors. Further studies characterizing Erk DNA binding with Mek and Erk inhibitors may help shed light on this hypothesis. Interestingly, Erk TKIs had a less pronounced effect on RTK reprogramming in the receptors studied here than Mek inhibition, which may be desired when considering a therapeutic option, yet cell proliferation was largely insensitive to Erk inhibition. Characterizing the RTK reprogramming in cells that are sensitive to Erk inhibition may help to understand if adaptation and efficacy are intrinsically connected within the MAPK pathway or if Erk inhibition maintains minimal effects on RTK reprogramming. An expansion of our knowledge of the molecular targets susceptible to RTK reprogramming may help characterize ideal targets within a pathway.

In addition to protein synthesis, the processes of endocytosis, degradation, and proteolytic shedding are largely decreased with Mek inhibition relative to control treatment and Erk inhibition. While these processes constitute post-synthesis mechanisms with respect to controlling RTK levels, it is plausible that the proteins governing these processes themselves are transcriptionally altered with MAPK inhibition. Interestingly, Mek has been shown to bind and phosphorylate heat shock factor 1 (HSF1), facilitating nuclear localization and transcription of heat shock proteins which are involved in a wide array of cellular processes including vesicular transport and protein degradation.^20^,^47^ As an effect upstream of Erk activity, HSF1 is an intriguing candidate protein for broadly affecting cellular processes such as endocytosis and degradation with Mek inhibition alone. As altered trafficking and degradation were a surprising outcome of the model predictions that spanned across RTKs, it will be important to continue to study these processes linked to drug resistance. As regulators of cellular homeostasis, characterizing the molecular players responsible for the adaptive response as well as identifying the extent that proteins not looked at in this study are affected by these processes will continue to expand our understanding of systems level changes with targeted therapeutics.

While BET inhibitors are being clinically evaluated as monotherapies, limited pre-clinical evidence shows benefit for their use in combination to overcome RTK reprogramming resistance.^45^ However, molecular understanding for rational combinations with BET inhibitors is currently limited. Although JQ1 was originally described to preferentially inhibit Myc transcriptional networks in multiple myeloma,^11^ new studies identify additional pathway effects for BET inhibition in alternate model systems.^2^,^43^ Furthermore, BET inhibitors not only inhibit transcription of BET activated genes, but they also induce transcription of BET repressed genes. As such, the molecular details of treatment with BET inhibitors or Mek inhibitors alone are not fully characterized and we have limited ability to predict combination treatments to attenuate RTK reprogramming and the subsequent resistance. To this point, both Mek and BET inhibitors have been described to affect transcriptional networks by inhibiting Myc, either by de-stabilization and protein degradation or reducing transcription and interrupting Myc-adaptor-chromatin interactions respectively. Yet the combination of the two has a larger anti-proliferative and anti-migratory response than either alone.

Furthermore, BET inhibition has no effect on Her2 levels when used alone but reduces Her2 levels in combination treatment with Mek inhibition, indicating Mek inhibitor induced BET dependency. Axl, however, is increased by both Mek inhibition and BET inhibition, retaining high levels with combination treatment such that cells are further inhibited by the addition of an Axl inhibitor. Following our hypothesis, this data indicates a Mek inhibitor induced loss of Erk transcriptional repression for Her2 and Axl whose newly active transcription is mediated in a BET dependent and independent manner respectively. Taken together, this indicates a lack of redundancy in the cellular targets and provides rationale for combining the two treatments, although relief of repressed transcriptional targets remains an issue for drug resistance. The suitability of these combinations will likely be context dependent and further study is needed to identify the governing rules.

Although our focus here is on RTK levels, there is an underlying assumption that these altered levels are indicative of increased signaling activity, promoting a cell survival response to MAPK inhibition. As Mek inhibition leads to increases in Axl and Her2 phosphorylation^13^,^34^ and we have found that increased Axl levels with Mek and BET inhibition correspond to a context dependent anti-proliferative effect of Axl inhibition, we believe our characterization of RTK levels is a suitable surrogate measurement. We have also limited our study to four RTKs and two cell lines. While these comparisons have enabled interesting insights regarding RTK specific responses, integrative mechanisms, and Mek vs. Erk inhibitor variable responses, studies of a larger scale of both proteins and cell lines will further the understanding and implications of these trends for improved selection of combination treatments. Fortunately, the combination of non-specificity of cell surface biotinylation and antibody mediated specificity for experimental measurements coupled to a generalized model structure grouping complex protein dependent processes (*i.e.,* ligand induced receptor dimerization/heterodimerization) into representative processes was purposeful to facilitate the adaptation to further proteins of interest without requiring detailed *a priori* understanding. A potential limitation of the lumped parameters, however, is that they may represent multiple underlying rates. For instance, EGFR and Her2, known heterodimerization partners, have been shown to internalize at different rates when homo- or heterodimerized,^22^ the combination of which will be captured with our model. The model and methodology could be extended to include a cross-linking protocol with different capture and detection antibodies for the heterodimerization partners to explicitly quantify the endocytosis rate to de-convolve the lumped parameter to provide better granularity if it were desired.

In summary, we have shown that using a model to extract mechanistic meaning from quantitative experiments allows us to understand the cellular processes altered by MAPK inhibition. We have further utilized model predictions to quantitate the effects of individual cellular process changes with inhibition, identifying multiple processes contributing to the RTK reprogramming phenotype. In doing so, we identified RTK dependent, integrative responses that vary with different MAPK target inhibitors. Taken together, the results propose a more complex picture of RTK reprogramming whereby no single mechanistic change, such as protein synthesis, alters RTK levels but there is a dynamic, integrative response. Increased understanding and accounting of this complexity will undoubtedly improve rational combination treatment selection to overcome resistance to targeted cancer therapies.

## Materials and Methods

### Cell Culture, Reagents, and Compounds

MDAMB231 cells were purchased from ATCC and grown in DMEM (Gibco) media supplemented with 10% FBS, 1% Pen/strep, and 1% Glutamax Supplement (Thermo Fisher) and maintained at 37 °C in 5% CO_2_. SUM159 cells were purchased from Asterand Bioscience and grown according to manufacturer’s suggestion. Recombinant human EGF was used at 10 nM. Batimastat (BB94, Tocris Bioscience) was used at 10 *μ*M. Selumetinib (AZD6244, Selleck Chem) was used at 1.5 *μ*M. Binimetinib (Mek162, ARRY-162, Selleck Chem) was used at 1.2 *μ*M. PD0325901 (Selleck Chem) was used at 33 nM. Ulixertinib (BVD-523, VRT752271, Selleck Chem) was used at 30 nM. DEL-22379 (Selleck Chem) was used at 5 *μ*M. GDC-0994 (Selleck Chem) was used at 30 nM. JQ1 (Tocris Bioscience) was used at 0.2 *μ*M. R428 (BGB324, Selleck Chem) was used at 1 *μ*M. DMSO at matched concentrations was used as all controls.

### Cell Lysis and Supernatant Collection

Prior to lysis, 200 *μ*L of the cellular supernatant was transferred to a 96 well v-bottom plate. Cells were lysed with 50 *μ*L NP40 lysis buffer (20 mM Tris–HCl, 150 mM NaCl, 2 mM EDTA, 1% NP40, 10% Glycerol, pH 7.4). Plates were shaken for 15 min at ~ 8000 rpm at 4 °C. Lysates were transferred to a 96 well v-bottom plate and lysates and supernatant samples were clarified by centrifugation for 15 min at ~ 2300×*g*. Samples were stored at − 20 °C prior to use.

### Luminex Reagents and Antibody-Bead Coupling

Capture antibodies for Axl, Met, EGFR, and Her2 were purchased from R&D Systems (MAB154, MAB3581, AF231, and MAB1129 respectively). Biotinylated detection antibodies for Axl, Met, EGFR and Her2 were purchased from R&D Systems (BAF154, BAF358, BAF231, and BAF1129 respectively). Streptavidin Phycoerythrin (SAPE) was purchased from Biorad (cat. no. 171304501). Assay diluent and washing buffer used in all steps was 0.1% BSA + 0.1% Tween20 in 1× PBS.

100 *μ*L MagPix beads (Luminex Corp.) were centrifuged at 10,000×*g* for 2 min and the supernatant was discarded. EDC (*N*-(3-dimethylaminopropyl)-*N*′-ethylcarbodiimide, Sigma) and S-NHS (*N*-hydroxysulfosuccinimide, Pierce) were dissolved in diH_2_0 to 50 mg/mL. Beads were incubated with 80 *μ*L activation buffer (100 mM NaH_2_PO_4_ pH 6.3), 10 *μ*L EDC, and 10 *μ*L S-NHS for 20 min dark at room temperature shaking at ~ 900 rpm. The mixture was centrifuged at 10,000×*g* for 2 min and the supernatant was discarded. Antibodies were diluted to 0.1 mg/mL in 100 *μ*L coupling buffer (50 mM HEPES, pH 7.4) and incubated with the bead suspension, overnight shaking, at 4 °C. The following day beads were washed 3× and resuspended in 1 mL 1% BSA + 1% Tween20 in 1x PBS and stored at 4 °C.

### Luminex Bead-Based ELISA Procedure

Capture antibodies were each diluted 75× into assay diluent (0.1% BSA + 0.1% Tween20 in 1× PBS) and 25 *μ*L was added to each well in a 384 well Optiplate (Perkin Elmer). The plate was briefly centrifuged at 200×*g* for 1 min and washed 3× with 80 *μ*L wash buffer (0.1% BSA + 0.1% Tween20 in 1× PBS). Samples were added to each well according to the following dilutions. End point measurement lysates were diluted at both 25× and 10× in assay diluent to a final volume of 25 *μ*L per well with data selected for further use based on location in the log-linear range of the standard curve. Supernatants were supplemented with 5% solution volume 0.1% BSA in 1× PBS to a final volume of 50 *μ*L. Standard curves were prepared at matched volumes and diluent concentrations as all samples. Samples were incubated with the capture antibody beads overnight shaking at ~ 8000 rpm at 4 °C. The following day the plate was centrifuged at 200×*g* for 1 min and washed 3× with 80 *μ*L wash buffer. Detection antibody was diluted 1000× into assay diluent and 15 *μ*L was added to each well. The plate was centrifuged at 200×*g* for 1 min, incubated at room temperature for 1 h, and washed 3× with 80 *μ*L wash buffer. SAPE was diluted 100× into assay diluent and 15 *μ*L was added to each well. The plate was centrifuged at 200×*g* for 1 min, incubated shaking at room temperature for 15 min, and washed 3× with 80 *μ*L wash buffer. After the final wash 60 *μ*L assay diluent was added to each well. Samples were read on a Flexmap 3D machine (Luminex Corp) according to manufacturer’s protocol.

### Time-Course Experiments

Cells were seeded at 50,000 cells/well and SUM159 cells were seeded at 18,750 cells/well in 48 well plates overnight. The following day cells were washed 1× with PBS and 125 *μ*L of 0.5 mg/mL Sulfo-NHS-SS-Biotin (Pierce) was added to each well and incubated for 1 h at 4 °C with gentle agitation. Cells were washed 1× with PBS followed by addition of treatment and incubated at 37 °C for 5, 20, 45, 75, or 90 min. Following incubation, cells were washed 1× with Reducing Buffer (50 mM Tris, 150 mM NaCl, pH 8.6) and cells were either stripped with the addition of 20 mM sodium 2-mercaptoethanesulfonate (MESNA, Sigma) or non-stripped with Reducing buffer and incubated for 1 h at 4 °C with gentle agitation. The stripping reaction was quenched by addition of 40 mM Iodoacetamide (IAA, Sigma) for 10 min at 4 °C with gentle agitation. Cells were washed 1× with PBS and lysed. Lysates were measured for total biotin signal (surface un-stripped with MESNA) and internal biotin signal (surface stripped with MESNA) with an adapted Luminex protocol, eliminating the detection antibody step. Independent experimental replicates were normalized to the mean of the measured total biotin signal at the first time point.

### End Point Experiments

MDAMB231 cells were seeded at 50,000 cells/well and SUM159 cells were seeded at 18,750 cells/well in 48 well plates overnight. The following day controls and treatments were added to 6 replicate wells for 24 h. Following treatment, supernatant and lysate were collected. Axl, Met, EGFR, and Her2 levels were simultaneously quantified in samples by bead-based ELISA (Luminex Corp) with recombinant protein standards (R&D systems).

### Absolute Receptor Quantification

End point measurements were converted from the measured mean fluorescence intensity (MFU) to absolute quantity (*m*_r_) in pg by five-parameter logistic regression to a recombinant protein standard. Subsequently, measurements were converted to receptor concentration (r, molecules/cell) through the following relationship:

1 $$r = \frac{{m_{\text{r}} \cdot dil \cdot {\text{prot}}_{\text{cell}} \cdot n_{\text{avg}} }}{{c_{\text{prot}} \cdot {\text{MW}}_{\text{RTK}} \cdot v_{\text{lys}} }}$$

where *dil* is the fraction of the total lysate and supernatant measured respectively, *c*_prot_ is the concentration of total protein in the lysate as measured by BCA assay, MW_RTK_ is the molecular weight of the recombinant protein standard, *v*_lys_ is the volume of lysate collected, *n*_avg_ is Avogadro’s number, and prot_cell_ is the protein content per cell, estimated here as 300 pg/cell as reported for HeLa cells.^29^

For absolute quantification, DMSO control treated samples were collected on three separate days (biological day replicates), on two separate plates each day (biological plate replicates), with 3 replicate wells per plate (technical plate replicates). Lysate and supernatant samples were collected and measured for MDAMB231 and SUM159 cells on 3 independent bead-based ELISA plates, with individually prepared reagents, including standard curves, on each plate. On plate standard curves were averaged and on plate samples were converted based on the mean standard curve. Absolute RTK quantification was calculated as the median RTK quantitation value (across biological day, plate and technical replicates) in a given condition (cell line, lysate vs. supernatant). For subsequent measurements, the offset was calculated as the fold change between the DMSO control in-experiment median value to the absolute quantitation value. This offset was applied to individual experimental points across all treatments, effectively shifting the experimental data to account for variations in the standard curve.

### Surface Fraction Assay

Cells were seeded overnight at the above described densities. The following day media was changed and cells were incubated at 37 °C for 24 h. Subsequently, cells were washed 1× with PBS and 125 *μ*L of 0.2 mg/mL cell permeable NHS-SS-Biotin (Pierce) was added to each well and incubated for 1 h at 4 °C with gentle agitation. Cells were then washed 1× with Reducing Buffer (50 mM Tris, 150 mM NaCl, pH 8.6) and cells were either stripped with the addition of 20 mM sodium 2-mercaptoethanesulfonate (MESNA, Sigma) or non-stripped with Reducing buffer and incubated for 1 h at 4 °C with gentle agitation. The stripping reaction was quenched by addition of 40 mM Iodoacetamide (IAA, Sigma) for 10 min at 4 °C with gentle agitation. Cells were washed 1× with PBS and lysed. Lysates were measured for total biotin signal (not stripped with MESNA) and internal biotin signal (surface stripped with MESNA) with an adapted Luminex protocol, eliminating the detection antibody step.

### Cell Viability Assay

MDAMB231 cells were seeded at 2500 cells/well and SUM159 cells were seeded at 2000 cells/well overnight in 96 well plates. The following day treatments were added and cells were incubated for 72 h at 37 °C and 5% CO_2_. Viability measurements were made using CellTiter-Glo Luminescent Cell Viability Assay (Promega) per manufacturer’s suggestions.

### Proliferation Assays

MDAMB231 cells were plated at 2500 cells/well in 96 well plates overnight. The following day treatments were added and cells were incubated for 100 h at 37 °C and 5% CO_2_. Plates were measured for percent confluence in the well using the IncuCyte Zoom System and Software (Essen Bioscience).

### Migration Assays

MDAMB231 cells were plated at 25,000 cells/well in 96 well plates overnight. The following day, a wound was made with a 96-well WoundMaker (Essen Bioscience) and wells were washed twice with 1× PBS. Treatments were added to wells and plates were measured for percent confluence in the wound using the IncuCyte ZOOM System and Software (Essen Bioscience) at 24-h post treatment.

### Cell Fixation, Staining, and Immunofluorescence Imaging

Cells were seeded onto 35 mm glass-bottom dishes with MDAMB231 cells at 100,000 cells/dish and SUM159 cells at 50,000 cells/dish overnight. On the second day treatments were spiked into the media for 4 h. Media was aspirated then cells were covered with 4% formaldehyde diluted in PBS at 37 °C for fixation. After 15 min at room temperature plates were rinsed in PBS. Cells were then covered with ice-cold 100% methanol for permeabilization. After 15 min at − 20 °C the methanol was aspirated and the plates rinsed in PBS. Blocking was performed in Odyssey Blocking Buffer (PBS) for 60 min and then the buffer aspirated. The primary antibodies used were diluted (1:400 p-p44/42 MAPK (Erk1/2) [Thr202/Tyr204] and p44/42 MAPK (Erk1/2) [L34F12]) in Odyssey Blocking Buffer (PBS) and incubated overnight at 4 °C. Cells were again rinsed in PBS. Fluorochrome-conjugated secondary antibodies used were diluted (1:200 Donkey anti-Mouse IgG Fluor 594, Donkey anti-Rabbit IgG Fluor 488) in Odyssey Blocking Buffer (PBS). DAPI was used to stain the nuclei for segmentation purposes (1:10,000). After 1 h at room temperature in the dark, cells were aspirated and rinsed in PBS before imaging. Imaging was performed on a Nikon Ti spinning disk Confocal Microscope with wide-field at 20x.

### Immunofluorescence Image Processing and Analysis

To analyze single-cell immunostaining of Erk and pErk, images were processed and analyzed as follows using in-house MATLAB scripts (Mathworks, Inc). Images were exported at the microscope-acquired bit depth using Nikon Elements software. Cell nuclei were first localized and segmented using point-source detection^1^ with a 2D Gaussian standard deviation of 6 pixels from the DAPI images, and segmented objects smaller than 42 *μ*m^2^ were empirically categorized as debris and removed. For cell body segmentation, the Erk immunostained images were used. The Erk-stained images were first retrospectively flat-field corrected to remove uneven illumination by first generating a flat-field image. This image was generated by averaging all Erk-stained images on a pixel-by-pixel basis and applying a 100 × 100 pixel median filter to the averaged image. Each Erk image was then divided on a pixel-by-pixel basis by the flat-field image. Cell bodies were then segmented using the flat-field corrected Erk-stained images by combining the overlap in foregrounds of intensity-based Otsu thresholding and Canny edge detection masks, followed by morphological closing of objects using a disk structural element of 2 pixels, and removal of all mask spur pixels. Putative cell body objects not overlapping with at least one pixel of the segmented nuclei mask were considered debris and removed. Touching cells were split from each other using a propagation algorithm^28^ part of the CellProfiler tool.^6^ Nuclei centers detected during the localization stage above were used as seeds to split nuclei objects using propagation with a regularization parameter of 5. Segmented and split nuclei were then used as seeds for the cell body mask, whereby cell bodies were split using propagation with a regularization parameter of 0 to fully utilize intensity variation in the objects to split touching cells. To analyze immunostaining signal intensities in the nucleus and cytoplasm of cells, local segmentation of nuclei was first performed by Otsu-based thresholding of the DAPI signal within each segmented cell body cropped image individually. The locally-segmented nuclear masks were eroded using a disk structural element of 2 pixels, to minimize the chance of including cytoplasm region pixels in the nuclei masks. The cytoplasm regions were defined as the cell body masks excluding the locally-segmented nuclei mask regions for each cell. For nucleus, cytoplasm, and whole-cell (nucleus + cytoplasm) compartments for each cell, mean pixel intensity was computed in the immunostained images.

### Model Structure

The model schematic is shown in Fig. 1 whereby receptors exist in 1 of 3 compartments: Cell surface (*r*_s_), intracellular (combined endosomal, lysosomal, nuclear) (*r*_i_), or freely circulating in the extracellular medium (*r*_f_). Movement between compartments is governed by the following processes: newly synthesized protein inserted to the cell membrane (*P*_syn_), protein degradation from the internal protein pool (lysosomal or proteasomal pathways) (*k*_deg_), proteolytic cleavage of protein ectodomains from the cell surface to freely circulating the supernatant (*k*_shed_), endocytosis from the cell surface to the internal compartment (*k*_end_), and recycling from the internal pool to the cell surface (*k*_rec_). These parameters are semi-mechanistic in that they lump more detailed processes, such as receptor-ligand binding and interaction with trafficking or degradation mediated proteins, to give an overview rate of the entire process. Based on mass action kinetics, we describe the system as:

2 $$\begin{array}{*{20}c} {\frac{{dr_{\text{i}} }}{dt} = k_{\text{end}} r_{\text{s}} - \left( {k_{\text{rec}} + k_{\text{deg}} } \right)r_{\text{i}} } \\ \end{array}$$

3 $$\begin{array}{*{20}c} {\frac{{dr_{\text{s}} }}{dt} = P_{\text{syn}} + k_{\text{rec}} r_{\text{i}} - \left( {k_{\text{end}} + k_{\text{shed}} } \right)r_{\text{s}} } \\ \end{array}$$

4 $$\begin{array}{*{20}c} {\frac{{dr_{\text{f}} }}{dt} = k_{\text{shed}} r_{\text{s}} } \\ \end{array}$$

with units of molecules cell^−1^ min^−1^ for *P*_syn_ and min^−1^ for *k*_deg_*, k*_end_*, k*_rec_, and *k*_shed_. As the end point experiments measure lysate and supernatant quantities, Eq. (4) is used to quantitate supernatant measures while *dr*_t_/*dt *=* dr*_i_/*dt *+* dr*_s_/*dt* is used to quantitate lysate measures. The time-course data is assumed to not measure protein synthesis as it is a pulse-chase experiment, altering Eq. (3) above to become

5 $$\begin{array}{*{20}c} {\frac{{dr_{\text{s}} }}{dt} = k_{\text{rec}} r_{\text{i}} - \left( {k_{\text{end}} + k_{\text{shed}} } \right)r_{\text{s}} } \\ \end{array}$$

Measured quantities in the time-course experiment are *r*_i_(*t*) (total labeled biotin internal to the cell with time) and *r*_t_(*t*) (total labeled biotin in the whole cell with time), where *dr*_t_/*dt *=* dr*_i_/*dt *+* dr*_s_/*dt*.

### Bayesian Inference for Parameter Estimation

In order to estimate the parameters for the model in Fig. 1, we simultaneously fit to both steady state and time-course data to capture the redundancy in parameters between data types. We undertake an approach that allows us to generate full parameter probability distributions, as opposed to a single best fit parameter set. Additionally, we incorporate expected values for a given parameter through setting a prior distribution as opposed to single literature-based values, allowing the results to reflect known quantitation for RTK mechanisms without constraining the result to any single RTK or condition. These goals are achieved using Bayes Theorem,

6 $$\begin{array}{*{20}c} {P\left( {\Theta |X} \right) = \frac{{P\left( {X|\Theta } \right)P\left( \Theta \right)}}{P\left( X \right)}} \\ \end{array}$$

Which states that the posterior probability distribution ($P\left( {\Theta |X} \right)$) is proportional to the product of the likelihood of the data ($P\left( {X|\Theta } \right)$) and the prior probability distribution ($P\left( \Theta \right)$) for a given parameter. $P\left( {\text{X}} \right)$ is the probability of the data which we do not calculate as $P\left( {\Theta |X} \right)$ is calculated as a ratio between step *i* and *i *+ 1. Operating in log space and assuming that log-transformed data is normally distributed, we calculate the data likelihood for any given parameter set as:

7 $$\begin{array}{*{20}c} {\log \left( {\text{Likelihood}} \right) = {\text{LL}} = \mathop \sum \limits_{e} \mathop \sum \limits_{t} \mathop \sum \limits_{r} - \frac{{\left( {\widehat{y}_{e,t} - y_{e,t,r} } \right)^{2} }}{{2\sigma_{e}^{2} }} - \log \left( {\sigma_{e} } \right) - \frac{1}{2}\log \left( {2\pi } \right) } \\ \end{array}$$

where $\widehat{y}$ is the simulated value, *y* is the experimental data, and LL values are summed over experiment type *e* (end point vs. time-course, supernatant vs. lysate, internal vs. total biotin), time points *t* (when applicable), and replicates *r*. Sigma ($\sigma )$ is the standard deviation of the data for experiment type *e*, where the median value from all cell lines, receptors, and treatments is used as a set value for any given experiment type.

### Prior Distribution Selection

The prior distribution for *k*_shed_ was approximated directly by the end point experimental data. Assuming *r*_s_ is time invariant, integrating Eq. (4) and rearranging identifies

8 $$\begin{array}{*{20}c} {k_{\text{shed}} = \frac{{r_{f} }}{{r_{t} f_{\text{s}} \Delta t}}} \\ \end{array}$$

where $\Delta t$ is the duration of the end point experiment and *f*_s_ is the protein surface fraction. Surface fraction was measured using a cell permeable, cleavable biotin linker (NHS-SS-Biotin). Based on this in-house data (Supplemental Fig. 10), *f*_s_ was modeled to have mean = 0.85 and standard deviation = 0.1 with boundary conditions at [0, 1]. Similarly, *r*_*f*_ and *r*_*s*_ have mean and standard deviation values from 12 replicate measures. Using 10,000 iterations, random values were drawn for *f*_s_, *r*_*f*_ and *r*_*s*_ from their associated normal distributions to calculate a data-based distribution for *k*_shed_. The distribution calculated for *k*_shed_ was then represented as a mean and standard deviation. As *r*_*s*_ was found to change with treatment, we evaluated an alternative extreme assumption whereby *r*_*t*_ linearly approaches its treatment induced end point measurement and numerically integrated Eq. (4) with a time-dependent *r*_*s*_. We found *k*_shed_ estimates indistinguishable from those calculated above, indicating *r*_*s*_ dynamics have a negligible effect on *k*_shed_ estimation.

As the Her2 supernatant measurement was undetected, the value *r*_*f*_ was instead represented by a uniform distribution from 1 to 89 molecules cell^−1^ h^−1^ and randomly sampled as for the distributions for measured data. The LLOQ was calculated to be 89 molecules cell^−1^ h^−1^ by finding the lowest point in the standard curve greater than 5 times the average background value and whose back-fit value was within 20% of the real value. From the resulting uniform distribution for Her2 *k*_shed_, minimum and maximum values were used as the lower and upper bounds of a uniform distribution used for the prior distribution.

With the exception of Her2 *k*_shed_, prior values were calculated assuming log transformed parameter values were normally distributed, such that

9 $$\begin{array}{*{20}c} {\log \left( {\text{Prior}} \right) = \mathop \sum \limits_{p} - \frac{{(\widehat{\mu }_{p} - \mu_{p} )^{2} }}{{2\sigma_{p}^{2} }} - \log \left( {\sigma_{p} } \right) - \frac{1}{2}\log \left( {2\pi } \right)} \\ \end{array}$$

where *p* are different parameters (*k*_deg_, *k*_end_, *etc*.), $\widehat{\mu }_{p}$ is the current test parameter value, $\mu_{p}$ is the prior distribution mean parameter value, and $\sigma_{p}$ is the prior distribution standard deviation. *k*_shed_ values for $\mu_{p}$ and $\sigma_{p}$ were as calculated above, whereas the other $\mu_{p}$ values were estimated from the literature to be as follows: 10^−2^ for *k*_deg_,^15^,^18^*k*_end,_^17^,^22^,^51^*k*_rec_^17^ and 10^2^ for *P*_syn_.^39^ Values were based on low end estimates as observed for EGFR as it is most commonly reported for the ligand stimulated case. $\sigma_{p}$ values were set to 10^2^ yielding wide priors, reflecting our lack of knowledge of the true parameter value for any given protein or condition.

Parameter distributions were first estimated for the control treated case, after which the prior distributions to be used for comparative treatments were updated to reflect the mean of these distributions. The control estimated parameter distribution standard deviation was expanded by twofold which we found optimal to maintain the goodness of fit to new data while minimizing parameter non-normality.

Non-parametric priors included surface fraction and initial conditions. Surface fraction values with mean = 0.85, standard deviation = 0.1 and boundary conditions at [0, 1] were used as for *k*_shed_ calculation. For end point data, initial supernatant concentrations, *r*_*f*,0_, were assumed to be zero due to media washout and initial lysate concentrations, *r*_*t*,0_, had a prior distribution representing the mean and standard deviation of the control treated case. Initial conditions for time-course data had mean values equal to that of the *t* = 5 time point with standard deviation values = 10 to allow for any dynamic changes between *t* = 0 and 5 min.

### Sequential Deterministic and Stochastic Optimization Implementation

We used a multi-step parameter estimation workflow to optimize global optimization as well as generate statistically based parameter distributions. First, we performed global optimization in Matlab (Mathworks) using the built-in *patternsearch* function using 100 parameter start sites generated using latin hypercube sampling. Upper and lower parameter bounds were set to [− 6,0] for *k*_deg_*, k*_end_*, k*_rec_, and *k*_shed_ and [0 5] for *P*_syn_, reflecting expansions on the prior distributions selected. The parameter set across all optimized start sites yielding the minimum objective function value was assumed to be the global optimum. Second, we performed local optimization for distribution calculation in Matlab (Mathworks) by altering the *mhsample* function to represent an adaptive metropolis algorithm^19^ for improved trace walk convergence. The adaptive metropolis function was run with initial covariance *C*_0_ = 0.01 for $t_{0} = 100$, scaling term *s*_d_= 0.2, and *ε* = 10^−20^. Initial start site was semi-randomly generated for 4 chains by drawing a random start site from a normal distribution with mean of the global minimum parameter value and 5% coefficient of variation and run for 100,000 iterations. Chain convergence was assessed visually and by Gelman Rubin convergence criteria 16 assuming random start sites within a local environment instead of for global optimization. The first 20,000 steps were discarded as the burn-in period.

### Predicted Lysate Levels

Control and treatment parameter distributions were reduced to mean values. Starting with the control treated parameter vector, each parameter mean for the treated case is individually substituted into the control vector and total lysate levels were simulated then normalized to the control treated lysate level to represent the treatment single parameter induced fold change.

### Principal Component Analysis (PCA)

PCA was performed in Matlab (Mathworks) using the *pca* function where the rows of the data block represent the cell lines and inhibitor treatments. Columns of the data block were performed in two different ways. First, the columns used were mean parameter distribution values for all parameters and all RTKs measured. Second, the columns used were the predicted lysate level changes for a given parameter and RTK. Unchanged variables were determined by randomly sampling 10% of parameter values from control and treated distributions, generating a difference distribution, and calculating the smaller of the fraction of samples above or below zero, representing a metric for distribution overlap. An arbitrary cutoff value was used such that distributions with more than 25% overlap with control were set to the control mean parameter value or set to predicted lysate fold change of one for the respective variable definition. Columns were z-score normalized across treatments for individual cell lines and then analyzed together to account for variations in absolute parameter magnitude across cell lines.

## Electronic supplementary material

Below is the link to the electronic supplementary material.

Supplementary material 1 (DOCX 15 kb)

Supplementary material 2 (EPS 2988 kb)

Supplementary material 3 (EPS 3065 kb)

Supplementary material 4 (EPS 3919 kb)

Supplementary material 5 (EPS 4059 kb)

Supplementary material 6 (EPS 2332 kb)

Supplementary material 7 (EPS 5235 kb)

Supplementary material 8 (EPS 5451 kb)

Supplementary material 9 (EPS 10761 kb)

Supplementary material 10 (PDF 84 kb)

Supplementary material 11 (EPS 2108 kb)

## Abbreviations

RTK: Receptor tyrosine kinase
BET: Bromodomain and extra-terminal domain
TNBC: Triple negative breast cancer
MAPK: Mitogen-activated protein kinase
TKI: Tyrosine kinase inhibitor
PCA: Principal component analysis
ELISA: Enzyme linked immunosorbent assay
MEKi: Mek inhibitor
BETi: BET inhibitor
AXLi: Axl inhibitor
LLOQ: Lower limit of quantitation
