# Supplementary material for: Systems Modeling Identifies Divergent Receptor Tyrosine Kinase Reprogramming to MAPK Pathway Inhibition
Source: Cell Mol Bioeng. 2018 Jul 26;11(6):451–69. doi: 10.1007/s12195-018-0542-y (PMC6244947; doi:10.1007/s12195-018-0542-y)
Supplement: Supplementary file 1 — Supplementary material 1 (DOCX 15 kb) [file 12195_2018_542_MOESM1_ESM.docx]

**Supplemental Figure 1. Experimental and modeling methodology capture EGF induced EGFR and Her2 endocytosis**

A) End point lysate and supernatant measurements across RTKs for MDAMB231 cells treated for 24 hours with 10 nM EGF. Data was normalized to the mean control treated value. Points indicate experimental data (n = 12) and shaded bars represent the range of simulated data from resampling 10% of parameter sets. * indicates p < 0.01 and **^..^** indicates p < 0.05 with a two-sample t-test after Bonferroni multiple hypothesis correction. B) Time-course measurements of internalized biotinylated protein across RTKs for MDAMB231 cells treated with 10 nM EGF for up to 90 minutes. Circles represent experimental replicates (n=18 for control, n = 6 for treatments), the dark lines represent the mean simulated values, and shaded areas indicates the range of simulated values from resampling 10% of parameter sets. C) *k_end_* parameter posterior distributions across RTKs, summed from 4 independent chains of 100,000 steps each with 20,000 burn-in period. The parameter *k_end_* has units of min^-1^. Black- control, blue- EGF.

**Supplemental Figure 2. Experimental and modeling methodology capture decreased Axl and Met shedding with batimastat**

A) End point lysate and supernatant measurements across RTKs for MDAMB231 cells treated for 24 hours with 10 μM batimastat. Data was normalized to the mean control treated value. Points indicate experimental data (n = 12) and shaded bars represent the range of simulated data from resampling 10% of parameter sets. * indicates p < 0.01 and **^..^** indicates p < 0.05 with a two-sample t-test after Bonferroni multiple hypothesis correction. B) Time-course measurements of total biotinylated protein across RTKs for MDAMB231 cells treated with 10 μM batimastat for up to 90 minutes. Circles represent experimental replicates (n=18 for control, n = 6 for treatments), the dark lines represent the mean simulated values, and shaded areas indicates the range of simulated values from resampling 10% of parameter sets. C) *k_shed_* parameter posterior distributions across RTKs, summed from 4 independent chains of 100,000 steps each with 20,000 burn-in period. The parameter *k_shed_* has units of min^-1^. Black- control, blue- batimastat.

**Supplemental Figure 3. Consistency of simulated versus experimental time-course data: total biotin signal**

Parameter sets were randomly sampled 32,000 times (representing 10% of parameter sets) and time-course data was simulated. Points represent mean +/- standard deviation of experimental replicates (n=18 for control, n = 6 for treatments), the dark line represents the mean simulated values, and shaded area indicates the range of simulated values. The y-axis is the signal normalized to the mean control treated total biotin measurement at t = 5 minutes and the x-axis is the time in minutes. Columns represent treatments (Sel- selumetinib, Bini- binimetinib, PD- PD0325901, Ulix- ulixertinib, DEL- DEL-22379, GDC- GDC-0994) and rows represent RTKs (Axl, Met, EGFR, Her2) for the two cell lines (MDAMB231 and SUM159) measured.

**Supplemental Figure 4. Consistency of simulated versus experimental time-course data: internal biotin signal**

Parameter sets were randomly sampled 32,000 times (representing 10% of parameter sets) and time-course data was simulated. Points represent mean +/- standard deviation of experimental replicates (n=18 for control, n = 6 for treatments), the dark line represents the mean simulated values, and shaded area indicates the range of simulated values. The y-axis is the signal normalized to the mean control treated total biotin measurement at t = 5 minutes and the x-axis is the time in minutes. Columns represent treatments (Sel- selumetinib, Bini- binimetinib, PD- PD0325901, Ulix- ulixertinib, DEL- DEL-22379, GDC- GDC-0994) and rows represent RTKs (Axl, Met, EGFR, Her2) for the two cell lines (MDAMB231 and SUM159) measured.

**Supplemental Figure 5. Viability with Mek and Erk inhibition in MDAMB231 and SUM159 cells.** Dose response curves as measured by CellTiterGlo assay at 72 hours (n = 6) for MDAMB231 cells (left panels) and SUM159 cells (right panels). (top panels) Mek inhibitors, blue- selumetinib, orange- binimetinib, green-PD0325901. (bottom panels) Erk inhibitors, blue- ulixertinib, orange- DEL-22379, green- GDC-0994. Points are normalized to the mean control treated value for each concentration and are shown as mean +/- standard deviation.

**Supplemental Figure 6. Mek inhibitors increase Axl and Her2 lysate levels and alter multiple parameter estimations.**

A) (left panel) End point lysate measurements and (right panel) end point percent shed (r_f_/r_t_) levels normalized to the mean control treated value for SUM159 cells treated for 24 hours with three Mek inhibitors. Points indicate experimental data (n = 12) and shaded bars represent the range of simulated data from resampling 10% of parameter sets. * indicates p < 0.01 and **^..^** indicates p < 0.05 with a two-sample t-test with Bonferroni multiple hypothesis correction. B) Parameter posterior distributions (summed for 4 independent chains, 100,000 iterations each, 20,000 step burn-in time) for control and Mek inhibitor treated cells and predicted protein level fold change relative to control treatment (red dots indicate observed treatment fold change). *P_syn_* has units of molecules cell^-1^ min^-1^ and *k_deg_, k_end_, k_rec_,* and *k_shed_* have units of min^-1^. Black- control, blue- selumetinib, orange- binimetinib, green- PD0325901.

**Supplemental Figure 7. Erk inhibitors have compound dependent parameter changes that vary from Mek inhibition.**

A) (left panel) End point lysate measurements and (right panel) end point percent shed (r_f_/r_t_) levels normalized to the mean control treated value for SUM159 cells treated for 24 hours with three Erk inhibitors. Points indicate experimental data (n = 12) and shaded bars represent the range of simulated data from resampling 10% of parameter sets. * indicates p < 0.01 and **^..^** indicates p < 0.05 with a two-sample t-test with Bonferroni multiple hypothesis correction. B) Parameter posterior distributions (summed for 4 independent chains, 100,000 iterations each, 20,000 step burn-in time) for control and Mek inhibitor treated cells and predicted protein level fold change relative to control treatment (red dots indicate observed treatment fold change). *P_syn_* has units of molecules cell^-1^ min^-1^ and *k_deg_, k_end_, k_rec_,* and *k_shed_* have units of min^-1^. Black- control, blue- ulixertinib, orange- DEL-22379, green- GDC-0994.

**Supplemental Figure 8. Model predicted lysate levels for randomly sampled parameter sets**

Lysate levels were predicted for the single parameter effect on treatment induced changes from control. 10,000 control and treated parameter sets were randomly sampled and lysate levels were predicted using the entire parameter set for control treatment and singly substituting treatment parameter values. Predicted lysate levels (r_t,prediction_) are shown as a fold change from control treatment. Columns represent treatments and rows represent RTKs (Axl, Met, EGFR, Her2) and cell lines (MDAMB231 and SUM159). Colors indicate single parameter effects for *k_deg_* (blue), *k_end_* (orange), *k_rec_* (green), *k_shed_* (purple), and *P_syn_* (grey). Two horizontal lines in the middle of the figure indicates a y-axis break, y-axis values not shown, to improve visualization of different distributions.

**Supplemental Figure 9. Mek inhibitors preferentially decrease Erk phosphorylation and nuclear localization.**

Immunofluorescence imaging of Erk, phospho-Erk (T202/Y204), and DAPI with different treatments. Scale bar indicates 100 $\mu m$.

**Supplemental Figure 10. Measured RTK surface fraction**

Total (r_t_) and internal (r_i_) protein pools were measured with a cell permeable, cleavable biotin linker without or with cell surface biotin stripping respectively in MDAMB231 cells after 24-hour control treatment (n=3). Surface fraction values were generated by calculating 1- r_i_/r_t_ pairwise for independent samples. Red cross indicates mean value.
