# Supplementary figures and images for: Systems Modeling Identifies Divergent Receptor Tyrosine Kinase Reprogramming to MAPK Pathway Inhibition
Source: Cell Mol Bioeng. 2018 Jul 26;11(6):451–69. doi: 10.1007/s12195-018-0542-y (PMC6244947; doi:10.1007/s12195-018-0542-y)

Supplemental Figure 9 Claas

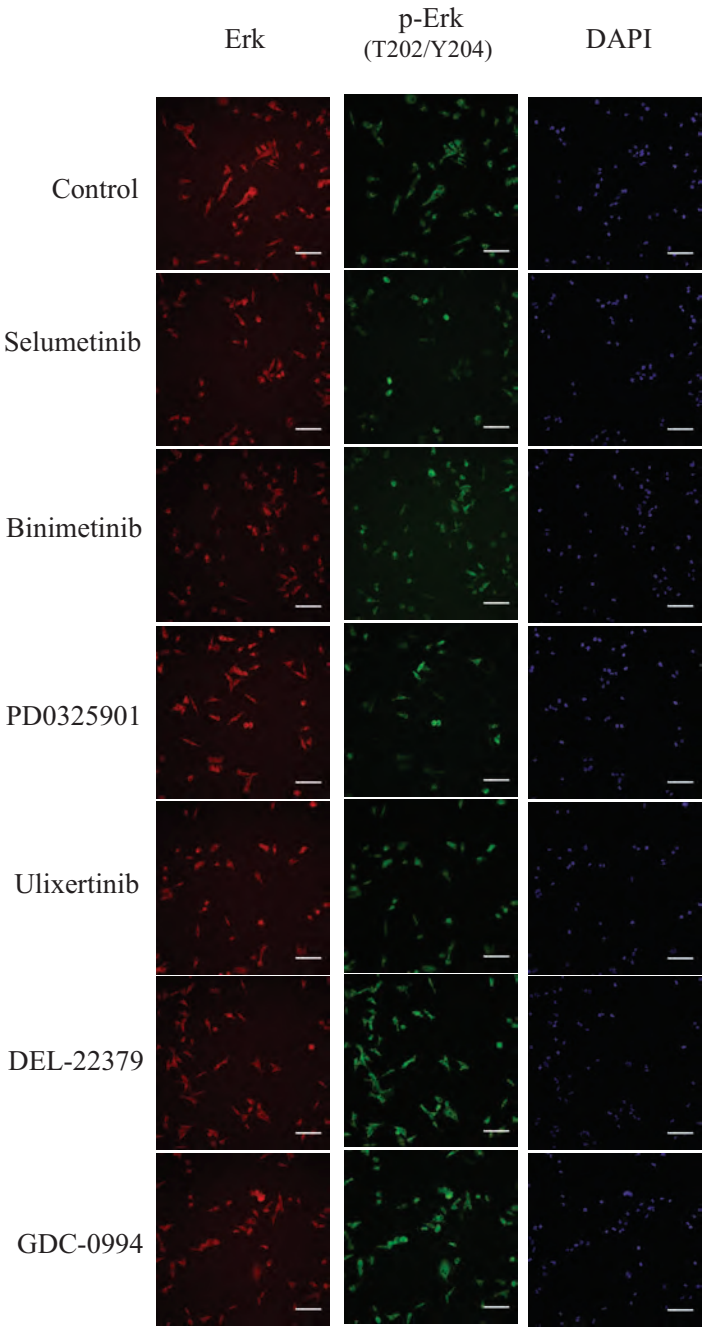

Supplement: Supplementary file 10 — Supplementary material 10 (PDF 84 kb) [file 12195_2018_542_MOESM10_ESM.pdf]
